# Delayed ureteric obstruction secondary to blood clot following renal biopsy: Case report and literature review

**DOI:** 10.1097/MD.0000000000042340

**Published:** 2025-05-02

**Authors:** Fenjuan Chen, Yiqi Huang, Meixiang Han, Jinying Wang

**Affiliations:** a Department of Nephrology, Shaoxing Second Hospital, Shaoxing, Zhejiang, China; b Department of Intensive Care Unit, Shaoxing Second Hospital, Shaoxing, Zhejiang, China.

**Keywords:** blood clot, case report, delayed, renal biopsy, ureteric obstruction

## Abstract

**Rationale:**

Hemorrhage is a frequent complication following renal biopsy, whereas ureteral obstruction secondary to blood clot is an uncommon but significant adverse event. Presently, such obstructions typically manifest within the first-week postbiopsy; however, there are no documented cases of delayed-onset ureteral obstruction secondary to blood clots.

**Patient Concerns:**

A 58-year-old male patient was admitted to Shaoxing Second Hospital with a 2-month history of recurrent bilateral lower extremity edema. He was diagnosed with nephrotic syndrome and subsequently underwent a renal biopsy. After a renal biopsy on day 19, the patient developed abdominal pain and hematuria. Follow-up examinations revealed a serum creatinine level of 181 μmol/L, and an abdominal computed tomography scan demonstrated a blood clot in the mid-portion of the right ureter.

**Diagnoses:**

The patient was diagnosed with ureteric obstruction and acute renal failure.

**Interventions:**

The patient initially underwent bladder irrigation therapy; however, as there was no observable improvement in the condition after 48 hours, the decision was made to perform a transurethral ureteric stent placement.

**Outcomes:**

The patient experienced immediate relief of abdominal pain following the procedure. On postoperative day 3, renal function had normalized. Seven days after surgery, the patient’s hematuria had completely resolved, and he was discharged from the hospital. Two weeks later, the ureteral stent was removed during an outpatient visit. During the 6-month follow-up period, the patient remained in excellent health with no complications.

**Lessons:**

This case represents the first documented instance of delayed secondary ureteral obstruction due to blood clots following renal biopsy in China. When symptoms such as hematuria and flank pain occur after the procedure, ureteric obstruction should be highly suspected. Early diagnosis and prompt treatment are critical for optimizing patient outcomes.

## 1. Introduction

Since its introduction by Iversen and Brun published in journal,^[1]^ The American Journal of Medicine, in 1951, renal biopsy has been recognized as the standard procedure for clinicians in diagnosing kidney diseases, formulating treatment plans, and assessing prognosis. With the advancement of biopsy equipment and real-time imaging technology, percutaneous renal biopsy has become a low-risk routine procedure in clinical practice.^[2]^ However, it may still result in complications that primarily present with hemorrhage,^[3,4]^ including retroperitoneal hematoma (11%), gross hematuria (3.5%), abdominal pain (4.3%), hemorrhage requiring red blood cell transfusion (1.6%), surgical intervention (0.01%), and death (0.008%). Ureteral obstruction secondary to blood clots is a rare complication following renal biopsy. To our knowledge, only 8 cases of ureteral obstruction secondary to blood clot following renal biopsy have been reported to date, with most occurring within 1 week post-biopsy.^[5–8]^ This article outlines the first documented case of delayed ureteral obstruction secondary to blood clot following renal biopsy in China, and we review and analyze the clinical features and management experiences of this rare complication as reported in the literature.

## 2. Case presentation

On May 23, 2024, a 58-year-old male with a documented history of diabetes mellitus for over a decade was admitted to the Nephrology Department of Shaoxing Second Hospital due to bilateral lower limb edema over the past 2 months. Upon admission, routine physical examination revealed body temperature of 36.9°C, blood pressure of 146/81 mm Hg, and bilateral pitting edema, without other significant abnormalities. The laboratory results are as follows: hemoglobin (Hb) 119 g/L, albumin 18 g/L, total cholesterol 8.2 mmol/L, serum creatinine (Scr) 87 μmol/L, calcium 1.86 mmol/L, urine protein 3+, urine red cells count 96.9 μL, urine protein/creatinine ≥ 300 mg/gCr, 24-hour protein excretion 7.58 g. Coagulation indicators were normal. The B-ultrasound assessment of the kidneys did not reveal any obvious size or morphological abnormalities.

The patient was initially diagnosed with nephrotic syndrome and subsequently underwent a renal biopsy for further evaluation. Following the renal biopsy, the patient exhibited pale pink gross hematuria and had a subsequent Hb level of 116 g/L. Hemostasis treatment was administered for 3 days. Pathological analysis of the renal biopsy confirmed a diagnosis of diabetic nephropathy, and the patient was advised to maintain strict glycemic control. Five days post-biopsy, the patient reported intermittent pale pink gross hematuria, with a urine red blood cell count of 354/μL, Hb level of 12 g/dL, and Scr of 86 μmol/L. Given the stabilization of the patient’s condition, he was deemed fit for discharge.

**Figure 1.**
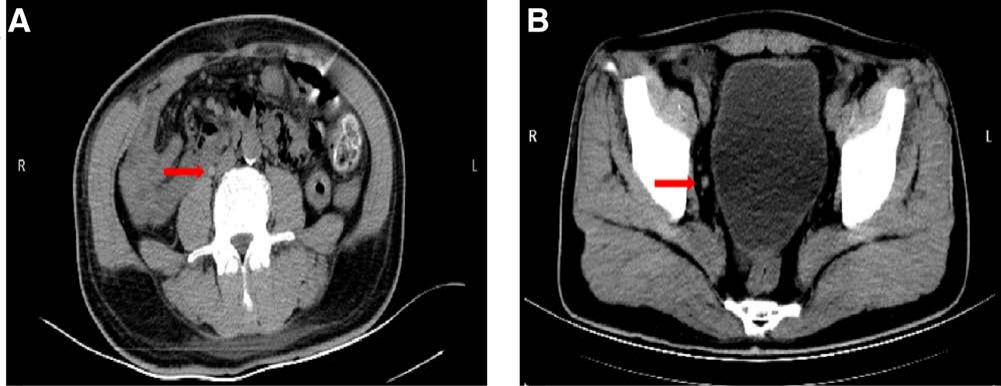
(A, B) The CT scan suggests that a blood clot in the right ureter. CT = computed tomography.

**Figure 2.**
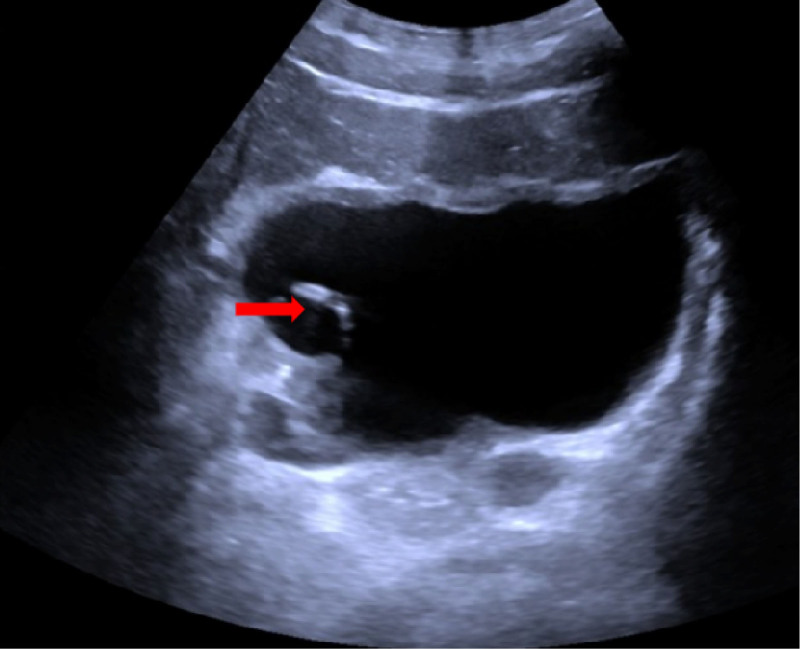
Ultrasound demonstrated a “J” stent in the right ureter and bladder, with no evidence of hematoma around the kidney or ureter.

**Figure 3.**
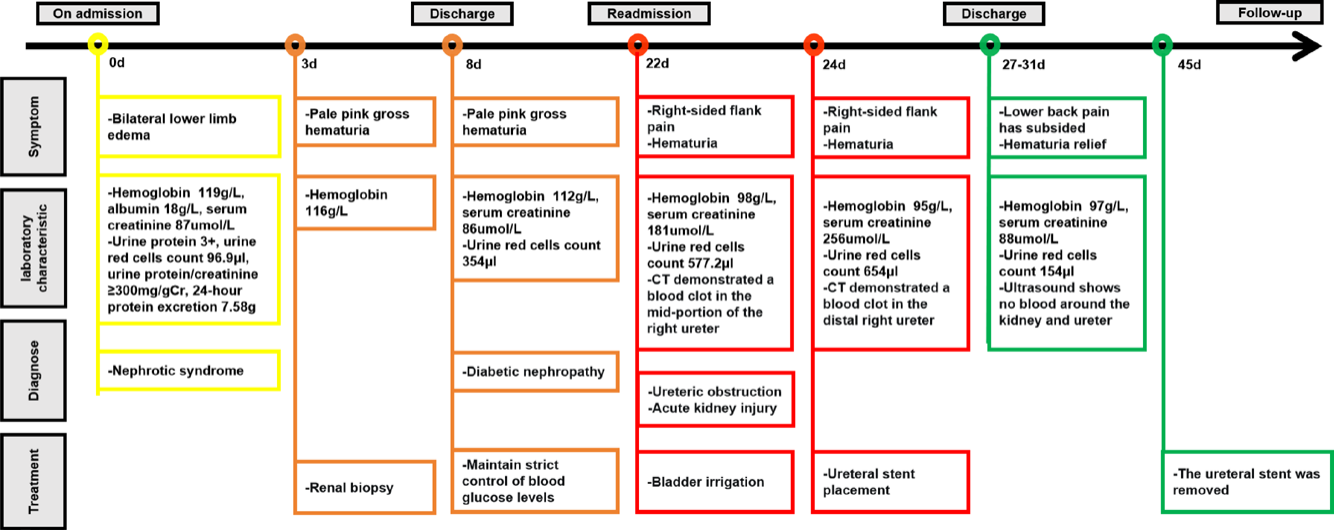
The entire process of diagnosis, treatment and outcome. CT = computed tomography.

After renal biopsy on day 19, the patient presented with right-sided flank pain and returned to our hospital for further evaluation. Laboratory tests revealed urine red blood cell count 577.2 μL, Hb 98 g/L, and Scr 181 μmol/L. An abdominal computed tomography scan demonstrated a small amount of perinephric hematoma around the right kidney and a blood clot in the mid-portion of the right ureter (Fig. 1A). The patient was diagnosed with ureteric obstruction and acute kidney injury and subsequently underwent bladder irrigation. Two days later, the patient experienced exacerbated right-sided abdominal pain and gross hematuria. Follow-up laboratory tests revealed urine red blood cell count 654 μL, Hb 95 g/L, and Scr 256 μmol/L. An abdominal computed tomography scan demonstrated a small amount of hemorrhage around the right kidney and psoas muscle and a blood clot in the distal right ureter (Fig. 1B). The attending physician reviewed the literature and determined that conservative treatment was ineffective, recommending immediate ureteral stent placement. The patient subsequently underwent transurethral ureteral stent insertion, resulting in immediate relief of abdominal pain. On postoperative day 3, laboratory tests revealed urine red blood cell count 154 μL, Hb 97 g/L, and Scr88 μmol/L. Urologic ultrasound demonstrated a “J” stent in the right ureter and bladder, with no evidence of hematoma around the kidney or ureter (Fig. 2). Seven days after surgery, the patient experienced complete resolution of hematuria and was discharged. Two weeks later, the ureteral stent was removed during an outpatient visit. During the 6-month follow-up, the patient did not experience any episodes of gross hematuria or abdominal pain. Figure 3 illustrates the timeline of diagnosis and treatment.

## 3. Discussion

Since the 1950s, renal biopsy has been established as a critical diagnostic tool in clinical practice for evaluating kidney diseases,^[1]^ although it is associated with a significant risk of complications. Beginning in the 1970s, advancements in biopsy equipment and real-time imaging techniques led to a marked reduction in both the incidence and severity of complications. Nonetheless, as an inherently invasive procedure, renal biopsy still carries the potential for post-procedural complications. A meta-analysis encompassing 39 studies involving 19,500 kidney biopsies showed that the overall complication rate in kidney biopsy cases was approximately 14.9%,^[9]^ with minor complications (non-clinically intervened transient hematuria, hematoma, and obvious pain at the biopsy site) and severe complications (such as transfusion, invasive radiology or surgery, acute renal obstruction, prolonged hospitalization, sepsis, or death) occurring at rates of 12.8% and 1.6%, respectively. Hemorrhage is the most common complication following renal biopsy.^[10]^ Minor hemorrhage typically manifests as hematuria or perinephric hematoma, while severe hemorrhage may necessitate blood transfusion, emergency angiography, or surgical intervention and can pose a significant risk to the patient’s life.^[10]^ This high incidence of hemorrhage complications is likely due to the kidneys’ unique anatomical and physiological characteristics: the renal blood flow accounts for approximately 1-quarter of cardiac output, and the deep retroperitoneal location of the kidneys makes compression hemostasis challenging.^[11]^

To the best of our knowledge, only 8 cases of ureteric obstruction secondary to blood clots following renal biopsy have been documented in the literature to date. These cases include 2 from Sweden, 1 from the United States, and 5 from Australia. In addition, 10 other retrospective studies reported adverse events of ureteral obstruction secondary to blood clots following renal biopsy. However, due to the absence of critical clinical details,^[12–18]^ such as age, gender, timing of diagnosis, intervention measures, and pathological findings from renal biopsy, we excluded these studies and included only cases with comprehensive data. This is the first reported case in China of ureteral obstruction secondary to a blood clot following renal biopsy. Unlike other cases, the ureteral obstruction in our patient presented with a delayed onset.

Table 1 summarizes the clinical data of ureteric obstruction secondary to blood clots following renal biopsy. The majority of patients were male, with an age range of 22 to 70 years. According to a meta-analysis by Corapi et al, which included 34 studies involving 9474 renal biopsy cases, female patients have a higher risk of post-biopsy hemorrhage.^[11]^ Luciano et al noted that younger patients tend to have a higher hemorrhage risk compared to elderly patients.^[19]^ However, our data present contrasting findings. This discrepancy may be attributed to the limited number of cases in our summary and the paucity of individual case reports. All patients, including our case, presented with hematuria. Other clinical manifestations included acute kidney injury (5/8), pain (4/8), hypotension (1/8), and septicemia (1/8). In nearly all cases (7/8), the interval from renal biopsy to diagnosis of ureteric obstruction was within 5 days. Only 1 case reported by O’Hara had a delay of 9 days, while our case presented with a delayed onset of 19 days, which is exceptionally rare. Upon reviewing the patient’s medical history, we found that the patient did not adhere to post-discharge rest instructions and engaged in daily activities such as shopping for food and climbing 6 flights of stairs. This noncompliance may have contributed significantly to the delayed hemorrhage following renal biopsy. Whittier et al found that 67% of major complications,^[20]^ including the need for blood transfusion or invasive surgery, acute renal failure, and death, occurred within 8 hours post-renal biopsy. Specifically, 91% of these events occurred within 24 hours, with the remaining 9% occurring after 24 hours. Another retrospective study involving 2138 patients undergoing renal biopsy reported that 91% of major complications occurred within 12 hours, 7.4% between 12 and 24 hours, and 1.85% after 24 hours.^[21]^ According to relevant guidelines,^[22]^ low-risk patients should be observed in the hospital for 6 to 8 hours following biopsy. In contrast, high-risk patients, characterized by severe renal dysfunction, acute kidney injury, pre-biopsy hypertension, age over 70 years, or the need for early anticoagulation therapy, should undergo an extended 24-hour observation period. In light of the delayed onset observed in this case, it is recommended to extend the duration of hospitalization for patients with poor compliance following renal biopsy. Additionally, healthcare providers should proactively arrange follow-up visits post-discharge to timely detect and manage any potential complications.

**Table 1:** Case summary of ureteral obstruction secondary to blood clot following renal biopsy.

References	Country	Gender	Age	Biopsy methodology	The duration from biopsy to diagnosis	Intervention	Biopsy pathology	Other manifestations	Prognosis
Stegmayr et al^[8]^	Sweden	Male	47	Percutaneous kidney biopsy	5 d	Streptokinase instillation	Mesangiocapillary glomerulonephritis	Haematuria, acute kidney injury and septicemia	Recovery
Grabe et al^[7]^	Sweden	Male	22	Percutaneous kidney biopsy	24 h	Ureteric stent insertion combined with pancreatic enzyme infusion	Mesangioproliferative glomerulonephritis	Suprapublic pain, hematuria and acute kidney injury	Recovery
Bergman et al^[6]^	United States	Male	41	Open kidney biopsy	Unknown	Ureteric stent insertion combined with streptokinase instillation	Minimal change glomerular disease with acute tubular necrosis	Acute renal colic and hematuria	Recovery
O’Hara et al case 1^[5]^	Australian	Unknown	60	Percutaneous kidney biopsy	5 d	Ureteric stent insertion	Non-specific mesangio-pathic changes	Haematuria and flank pain	Recovery
O’Hara et al case 2^[5]^	Australian	Unknown	70	Percutaneous kidney biopsy	9 d	Ureteric stent insertion	Diabetic and hypertensive nephropathy	Haematuria and flank pain	Recovery
O’Hara et al case 3^[5]^	Australian	Unknown	60	Percutaneous kidney biopsy	1 h	Ureteric stent insertion	IgA nephropathy	Hematuria, hypotension and acute kidney injury	Recovery
O’Hara et al case 4^[5]^	Australian	Unknown	60	Percutaneous kidney biopsy	3 d	Ureteric stent insertion	Focal necrotizing glomerulonephritis	Hematuria and acute kidney injury	Recovery
O’Hara et al case 5^[5]^	Australian	Unknown	60	Percutaneous kidney biopsy	<24 h	Percutaneous nephrostomy	Moderate glomerulitis, C4D negative, moderate interstitial and tubular fibrosis	Hematuria and acute kidney injury	Death
Present report (2024)	China	Male	58	Percutaneous kidney biopsy	19 d	Ureteric stent insertion	Diabetic nephropathy	Haematuria, flank pain and acute kidney injury	Recovery

Currently, the guidelines address the management of hemorrhage following renal biopsy but do not adequately describe ureteral obstruction caused by blood clots.^[23]^ Considering that ureteral obstruction represents a severe and time-critical complication demanding urgent intervention, it is imperative to incorporate comprehensive strategies addressing this issue into renal biopsy guidelines, thereby enhancing the awareness and preparedness of nephrologists. Regarding the treatment of ureteric obstruction, the primary approaches include surgical intervention (ureteric stent insertion [6/8], percutaneous nephrostomy [6/8]) and pharmacological dissolution (streptokinase instillation [2/8], pancreatic enzyme infusion [1/8]). In this case, after conservative treatment failed, we conducted a literature review that showed all reported cases required additional interventions. We immediately chose ureteric stent insertion. It is evident that case reports can provide positive assistance to clinicians when they encounter difficult diseases.

The pathological types include diabetic nephropathy, IgA nephropathy, mesangiocapillary glomerulonephritis, mesangioproliferative glomerulonephritis, acute tubular necrosis, and focal necrotizing glomerulonephritis. Establishing a link between these types and post-biopsy hemorrhage or ureteral obstruction is challenging, likely due to the limited number of cases. Previous studies have demonstrated that patients with acute tubular necrosis, IgA nephropathy, and lupus nephritis exhibit a significantly higher risk of post-biopsy hemorrhage compared to other pathological types.^[24,25]^ This increased risk may be attributed to the propensity of these conditions to cause glomerulosclerosis and interstitial fibrosis. Furthermore, while ureteral obstruction secondary to biopsy-induced thrombi generally has a favorable prognosis, 1 case resulted in death due to cardiac arrest. These findings underscore the critical importance of timely intervention.

## 4. Conclusion

In summary, renal biopsy has evolved into a standard clinical procedure for nephrologists. Its safety profile has been significantly enhanced by advancements in biopsy equipment and real-time imaging technology. However, serious complications such as ureteral obstruction secondary to post-biopsy thrombosis remain a concern and require vigilant monitoring. This case represents the first reported instance of delayed secondary ureteral obstruction due to post-biopsy blood clots in China. Due to the limited number of reported cases, there is no uniform treatment protocol for this complication. However, with early diagnosis and timely treatment, the overall prognosis of patients is good.

## Author contributions

**Conceptualization:** Fenjuan Chen, Meixiang Han, Jinying Wang.

**Data curation:** Fenjuan Chen, Yiqi Huang, Meixiang Han, Jinying Wang.

**Formal analysis:** Fenjuan Chen, Meixiang Han, Jinying Wang.

**Funding acquisition:** Fenjuan Chen, Jinying Wang.

**Investigation:** Jinying Wang.

**Supervision:** Yiqi Huang, Jinying Wang.

**Validation:** Fenjuan Chen, Yiqi Huang, Jinying Wang.

**Visualization:** Fenjuan Chen, Yiqi Huang, Jinying Wang.

**Writing – original draft:** Fenjuan Chen, Yiqi Huang, Meixiang Han, Jinying Wang.

**Writing – review & editing:** Fenjuan Chen, Jinying Wang.

## References

[1] Iversen P Brun C. Aspiration biopsy of the kidney. Am J Med. 1951;11:324–30. 14877837 10.1016/0002-9343(51)90169-6

[2] Pakfetrat M Malekmakan L Hamidianjahromi A Rastegar R. Frequency and timing of renal biopsy complications. Caspian J Intern Med. 2024;15:96–100. 38463932 10.22088/cjim.15.1.10 PMC10921104

[3] Poggio ED Mcclelland RL Blank KN.. Systematic review and meta-analysis of native kidney biopsy complications. Clin J Am Soc Nephrol. 2020;15:1595–602. 33060160 10.2215/CJN.04710420 PMC7646247

[4] Varnell CD Jr Stone HK Welge JA. Bleeding complications after pediatric kidney biopsy: a systematic review and meta-analysis. Clin J Am Soc Nephrol. 2019;14:57–65. 30522995 10.2215/CJN.05890518 PMC6364534

[5] O’Hara DV Wong JK Cooper B Wong G Wong MG Cheikh Hassan HI. Lessons for the clinical nephrologist: ureteric obstruction secondary to blood clot after kidney biopsy. J Nephrol. 2021;34:2131–6. 33856685 10.1007/s40620-021-01012-2 PMC8610936

[6] Bergman SM Frentz GD Wallin JD. Ureteral obstruction due to blood clot following percutaneous renal biopsy: resolution with intraureteral streptokinase. J Urol. 1990;143:113–5. 2294237 10.1016/s0022-5347(17)39884-1

[7] Grabe M Forsberg B. Retrograde trypsin instillation into the renal pelvis for the dissolution of obstructive blood clots. Eur Urol. 1986;12:69–70. 3948901 10.1159/000472581

[8] Stegmayr B Orsten PA. Lysis of obstructive renal pelvic clots with retrograde instillation of streptokinase. A case report. Scand J Urol Nephrol. 1984;18:347–50. 6505652 10.3109/00365598409180209

[9] Kajawo S Ekrikpo U Moloi MW. A systematic review of complications associated with percutaneous native kidney biopsies in adults in low- and middle-income countries. Kidney Int Rep. 2021;6:78–90. 33426387 10.1016/j.ekir.2020.10.019 PMC7783578

[10] Kajawo S Moloi MW Noubiap JJ Ekrikpo U Kengne AP Okpechi IG. Incidence of major complications after percutaneous native renal biopsies in adults from low-income to middle-income countries: a protocol for systematic review and meta-analysis. BMJ Open. 2018;8:e020891. 10.1136/bmjopen-2017-020891 PMC5922518 29703858

[11] Corapi KM Chen JL Balk EM Gordon CE. Bleeding complications of native kidney biopsy: a systematic review and meta-analysis. Am J Kidney Dis. 2012;60:62–73. 22537423 10.1053/j.ajkd.2012.02.330

[12] Birnholz JC Kasinath BS Corwin HL. An improved technique for ultrasound guided percutaneous renal biopsy. Kidney Int. 1985;27:80–2. 3981874 10.1038/ki.1985.13

[13] Tsai SF Chen CH Shu KH. Current safety of renal allograft biopsy with indication in adult recipients: an observational study. Medicine (Baltimore). 2016;95:e2816. 26871853 10.1097/MD.0000000000002816 PMC4753949

[14] Boschiero LB Saggin P Galante O. Renal needle biopsy of the transplant kidney: vascular and urologic complications. Urol Int. 1992;48:130–3. 1585505 10.1159/000282315

[15] Chan R Common AA Marcuzzi D. Ultrasound-guided renal biopsy: experience using an automated core biopsy system. Can Assoc Radiol J. 2000;51:107–13. 10786919

[16] Wilczek HE. Percutaneous needle biopsy of the renal allograft. A clinical safety evaluation of 1129 biopsies. Transplantation. 1990;50:790–7. 2238054 10.1097/00007890-199011000-00010

[17] Schmid A Jacobi J Kuefner MA. Transvenous renal transplant biopsy via a transfemoral approach. Am J Transplant. 2013;13:1262–71. 23489636 10.1111/ajt.12199

[18] Mcdonald MW Sosnowski JT Mahin EJ Willard DA Lamm DL. Automatic spring-loaded biopsy gun with ultrasonic control for renal transplant biopsy. Urology. 1993;42:580–2. 8236603 10.1016/0090-4295(93)90280-n

[19] Luciano RL Moeckel GW. Update on the native kidney biopsy: core curriculum 2019. Am J Kidney Dis. 2019;73:404–15. 30661724 10.1053/j.ajkd.2018.10.011

[20] Whittier WL Korbet SM. Timing of complications in percutaneous renal biopsy. J Am Soc Nephrol. 2004;15:142–7. 14694166 10.1097/01.asn.0000102472.37947.14

[21] Prasad N Kumar S Manjunath R. Real-time ultrasound-guided percutaneous renal biopsy with needle guide by nephrologists decreases post-biopsy complications. Clin Kidney J. 2015;8:151–6. 25815170 10.1093/ckj/sfv012 PMC4370312

[22] MacGinley R Champion De Crespigny PJ Gutman T. Kha-cari guideline recommendations for renal biopsy. Nephrology (Carlton). 2019;24:1205–13. 31490584 10.1111/nep.13662

[23] Hogan JJ Mocanu M Berns JS. The native kidney biopsy: update and evidence for best practice. Clin J Am Soc Nephrol. 2016;11:354–62. 26339068 10.2215/CJN.05750515 PMC4741037

[24] Peters B Stegmayr B Andersson Y Hadimeri H Molne J. Increased risk of renal biopsy complications in patients with iga-nephritis. Clin Exp Nephrol. 2015;19:1135–41. 25951807 10.1007/s10157-015-1121-3

[25] Chen TK Estrella MM Fine DM. Predictors of kidney biopsy complication among patients with systemic lupus erythematosus. Lupus. 2012;21:848–54. 22415926 10.1177/0961203312439334 PMC3767126

